# Supplementary material for: The intake of ultra-processed foods, all-cause, cancer and cardiovascular mortality in the Korean Genome and Epidemiology Study-Health Examinees (KoGES-HEXA) cohort
Source: PLoS One. 2023 May 4;18(5):e0285314. doi: 10.1371/journal.pone.0285314 (PMC10159145; doi:10.1371/journal.pone.0285314)
Supplement: S1 Table — (DOCX) [file pone.0285314.s001.docx]

# S1 Table. Classification of FFQ food items according to the NOVA classification

| NOVA group | HEXA Food group on the FFQ | Examples of food items/recipe items |
| --- | --- | --- |
| 1) Unprocessed or minimally processed foods | White rice; multi-grain rice; mixed-grain rice; bean rice; mixed rice (half bean half rice); rice cake | White rice, buckwheat, soybean, black bean, black pollack, seoritae, *Gijang*, fermented soybean, sorghum, tea water, *Yulmu* rice, glutinous rice, glutinous rice cake, tteok, rice balls |
|  | **Dumpling** | Tofu*, flour*, pork*, tenderloin*, boiled bean sprouts*, raw eggs* |
|  | **Black Bean Noodles/Spicy Seafood Noodles** | Raw onions*, pork*, raw carrots*, beans*, raw mushroom*, raw garlic*, raw squid*, mussels* |
|  | **Peanuts/almonds/Pine nuts** | Dried peanut*, roasted peanuts*, roasted pine nuts* |
|  | Beans | Soybean, black bean, black pollack; dried yellow bean, soy sauce |
|  | **Fermented soybean paste** | Fermented soybean* |
|  | eggs/quail eggs |  |
|  | **Tofu**; “*muk*” | Tofu; acorn jelly, Mung-bean cake, buckwheat cake |
|  | Potatoes and sweet potatoes | Raw potatoes, raw sweet potatoes |
|  | Radish; cabbage/cabbage soup; spinach; lettuce; perilla leaves; vegetable wraps/salads; other green vegetables; bellflower/duck; bean sprouts/host sprouts; bracken/sweet potato stems; oyster mushrooms; other mushrooms; red pepper leaves/chive/*minari*; green onion/pumpkin juice; pumpkin |  |
|  | **Carrot/carrot juice** | Raw carrot* |
|  | Grilled pork belly; Grilled pork/Stir-fried/broiled; Steamed pork; By-products (beef entrails, blood pudding, blood sausage); steak/roasted beef (ribs, sirloin, tenderloin, *bulgogi*; dog meat; fried chicken/ chicken soup/ *samgyetang*/ chicken soup; soups (beef soup, *yukgaejang*, etc.) |  |
|  | Raw fish | Flounder, Tuna, sea bass |
|  | Raw mackerel / saury; hairtail; eel; corvina / sea bream/flounder |  |
|  | Pollack, dried; squid/octopus, dried; anchovies, stir-fried | Raw Octopus, raw flounder, raw pollack, frozen pollack, raw octopus, raw squid, raw anchovies |
|  | clam/snail (including soup, stew, grilled, chopped noodles, seasoned, etc.) | Raw crabmeat, ramie clam, raw lily, raw clam, raw blood clam, raw mussels |
|  | Oyster; Crab; Shrimp | Oyster, crab, sesame, shrimp(boiled, raw, fried) |
|  | Seaweed | laver, raw seaweed, raw kelp |
|  | **Milk** | Plain milk |
|  | Coffee; green tea | Coffee powder, instant; green tea |
|  | strawberry; melon/melon; watermelon; peach/plum; banana; persimmon/dried; tangerine; pear; apple; orange/orange juice; grape/wine juice; **tomato/tomato ketchup** | Strawberry; melon musk; melon; watermelon, raw; plum; peach, raw; banana, raw; persimmon, tangerine, raw; kumquat, raw; stone pear; pear, raw; apple, raw, apple juice, grape juice; tomato juice |
| 2) Processed culinary ingredients | **Jam/Honey/Butter/Margarine (if eaten on bread)** | Honey* |
|  | **Black Bean Noodles/Spicy Seafood Noodles** | Black soybean sauce*, soybean oil*, green pepper powder* |
|  | Grain powder | *Deodeok* root powder*, lactic acid bacteria* |
|  |  |  |
|  | Table sugar; coffee cream | Table sugar, coffee cream**^a^** |
|  | **Dumplings** | soybean oil* |
| 3) Processed foods | **peanuts/almonds/Pine nuts** | Seasoned almonds* |
|  | Korean spaghetti | Boiled noodles, boiled *Udon* |
|  | **Black Bean Noodles/Spicy Seafood noodles** | Boiled chinese noodles*, potato powder* |
|  | Cold noodles | Raw buckwheat noodles |
|  | Glass noodles/potato noodles |  |
|  | Fermented soybean paste | Mixed paste (*ssamjang*) * |
|  | **Tofu** | Soft tofu* |
|  | Pickled vegetables | Pickled red pepper, Pickled radish, Pickled garlic |
|  | Grain powder/pre-meal (ready to eat porridges)^a^ | barley, powder; sesame, black sesame, roasted; kelp, dried; carrot, raw; minari; *Myeongil* leaf; radish; Seaweed, powder; white rice; sorghum; mugwort, raw; lotus root; mushroom, raw; burdock, raw; *yulmu* rice; kale; *shiitake* mushroom, oak, dried; dried pumpkin |
|  | corvina / sea bream / crabmeat; Pollack; dried squid / octopus; anchovies / stir-fried anchovies; tuna-can; salted fish; fishcake / crabmeat; seaweed / kelp | Tuna-can; anchovy, salted; pollack, salted; yellowtail, salted; sea urchin, salted; squid; salted; shellfish; shrimp, salted, fried fish cake; dried seaweed |
|  | Cheese | Processed cheese; mozallera cheese^a^ |
|  | **Other drinks** | Citron tea*, Ginseng tea*, *Ssanghwa* tea* |
|  | carrot/carrot juice; peach/plum; apple; orange/orange juice; grape/wine juice; **tomato/tomato juice/tomato ketchup** | carrot can juice; peach, canned; orange, canned juice; tomato, canned tomato; tomato, tomato paste, canned tomato; tomato puree |
|  | Fermented vegetables (*Kimchi*) | Cabbage kimchi, radish kimchi, *Nabak* kimchi/*Dongchimi* and other kimchi |
| 4) Ultra-processed foods | Instant noodles (*ramen*) | Instant noodles |
|  | cornflakes | cereal, rice crisps; cereal cornflakes; cereal, brown rice flakes |
|  | Loaf bread (‘*sikppang*’); bread with red bean; other bread** | Bread, red bean bread, buns, red bean filling, *gombo* bread; doughnut; cream bread; castella |
|  | **Jam/honey/butter/margarine (when eaten on bread)** | strawberry jam*, apple jam*, apricot jam*, grape jam*, butter*, margarine* |
|  | Cake/*chocopie;* cookie/cracker/snack; candy/chocolate | Choco Pie, Roll Cake, Whipped Cream, Blueberry Cake, Sponge Cake, Pound Cake, Hot Cake/Pancake, Montshell /Fresh cream; Dry Bread, Biscuit, Soft, Sen Bay, Rice Cake, Potato chips/crisps, Shrimp Snack, Corn snacks, Crackers; Drops, Candy |
|  | Pizza/hamburger | Pizza, hamburger |
|  | Processed meat (ham, sausage) | Raw bacon, raw Sausage, dry Sausage, Frankfurt Sausage, Hot Dog, Ham, Slice, Luncheon Meat |
|  | Fish cake/Crab meat | Imitation crab*, crab sausage* |
|  | **milk** | Coffee milk*, chocolate milk*, fruit flavored milk* |
|  | yogurt/*Yoplait**** | Liquid yoghurt, strawberry-flavored yoghurt |
|  | Ice-cream | Ice cream, 12% fat; ice cream, 8% fat; ice cream, strawberry-flavored; ice cream, vanilla flavored; ice cream, chocolate-flavored; sherbet; selection; world corn; Joanna |
|  | Soymilk drink | Soymilk drink (*vegemil*) |
|  | Soft drinks (cola/cider)/ fruit beverages | fruit soda; lemon soda; orange soda; cream soda; grape soda; cider; coke |
|  | **Other drinks** | Sweet rice punch (*Sikhye*) * |

*Weighted amounts from mixed dishes

**The loaf bread *(‘sikppang’*) /toast bread /buns consumed in Korea are mainly mass produced, packaged, contains additives, and commonly sold in convenience stores and marts. The SQFFQ mainly assessed the commonly consumed breads in Korea

***The major brand of yoghurt consumed in korea, and assessed in the SQFFQ was the liquid variety called ‘*Yoplait*’. Yoplait yoghurt is sweetened, flavored, colored and has artificial additives according to the labelling information.
