# Supplementary material for: The intake of ultra-processed foods, all-cause, cancer and cardiovascular mortality in the Korean Genome and Epidemiology Study-Health Examinees (KoGES-HEXA) cohort
Source: PLoS One. 2023 May 4;18(5):e0285314. doi: 10.1371/journal.pone.0285314 (PMC10159145; doi:10.1371/journal.pone.0285314)
Supplement: S2 Table — (DOCX) [file pone.0285314.s002.docx]

# S2 Table. Characteristics of participants according to quartiles of ultra-processed food intake

|  |  |  | **Quartiles of UPF intake, % food weight** | | | | | | | |
| --- | --- | --- | --- | --- | --- | --- | --- | --- | --- | --- |
|  |  |  | **Men** |  |  |  |  | **Women** |  |  |
| **Characteristic** | | **Q1** | **Q2** | **Q3** | **Q4** |  | **Q1** | **Q2** | **Q3** | **Q4** |
| Participants, n | | 9711 | 9712 | 9712 | 9712 |  | 18,682 | 18,682 | 18,683 | 18,682 |
| Age, years |  | 56.2±0.1 | 53.8±0.1 | 52.8±0.1 | 52.1±0.1 |  | 54.9±0.1 | 52.1±0.1 | 51.5±0.1 | 51.2±0.1 |
| Age group | 40-49 | 21.3 | 33.1 | 37.5 | 41.8 |  | 25.6 | 38.8 | 41.7 | 43.7 |
|  | 50-59 | 40.1 | 38.6 | 36.5 | 34.6 |  | 43.8 | 41.6 | 41.2 | 40.1 |
|  | 60-69 | 38.6 | 28.3 | 26.0 | 23.7 |  | 30.6 | 19.6 | 17.1 | 16.1 |
| Education | Elementary | 11.9 | 8.6 | 6.9 | 6.2 |  | 28.3 | 17.7 | 13.6 | 12.3 |
|  | Middle | 15.6 | 12.6 | 11.1 | 9.8 |  | 21.5 | 18.2 | 16.6 | 15.0 |
|  | High school | 39.4 | 38.6 | 36.5 | 35.6 |  | 36.3 | 42.2 | 43.2 | 43.4 |
|  | ≥College | 33.2 | 40.2 | 45.6 | 48.5 |  | 14.0 | 21.9 | 26.6 | 29.3 |
| Income, USD | Unknown | 8.4 | 6.8 | 6.0 | 6.3 |  | 10.8 | 8.8 | 8.6 | 10.2 |
|  | <1000 | 9.5 | 7.2 | 5.6 | 5.3 |  | 16.3 | 10.3 | 8.5 | 8.1 |
|  | 1000~3000 | 42.9 | 40.1 | 37.6 | 37.3 |  | 41.8 | 40.1 | 39.2 | 37.2 |
|  | ≥3000 | 39.3 | 46.0 | 50.7 | 51.1 |  | 31.2 | 40.9 | 43.6 | 44.5 |
| Married/cohabiting | | 95.2 | 94.8 | 94.6 | 92.6 |  | 86.2 | 88.4 | 88.3 | 86.2 |
| Residence | Seoul/ Metropolitan | 34.0 | 36.6 | 38.1 | 39.6 |  | 30.5 | 33.3 | 35.7 | 37.5 |
|  | Metropolitan cities | 32.8 | 28.5 | 28.5 | 28.8 |  | 39.6 | 35.9 | 35.0 | 33.2 |
|  | Others | 33.2 | 34.8 | 33.4 | 31.6 |  | 29.9 | 30.8 | 29.3 | 29.3 |
| Smoking | Current smoker | 42.7 | 43.0 | 40.2 | 38.7 |  | 0.9 | 1.2 | 1.3 | 1.6 |
|  | Past smoker | 30.1 | 31.2 | 31.7 | 32.3 |  | 2.1 | 2.2 | 2.2 | 2.7 |
| Drinking | Current drinker | 74.3 | 74.2 | 72.3 | 70.2 |  | 25.1 | 32.5 | 32.6 | 33.0 |
|  | Past drinker | 6.6 | 7.1 | 7.2 | 7.4 |  | 1.7 | 1.7 | 1.9 | 2.1 |
| AMI |  | 1434±40 | 1342±39 | 1408±39 | 1360±38 |  | 1599±14 | 1582±14 | 1633±14 | 1599±13 |
| Regular physical exercise | | 36.8 | 35.3 | 36.1 | 35.2 |  | 34.9 | 35.0 | 36.5 | 37.3 |
| BMI, kg/m2 | | 24.4±0.0 | 24.4±0.0 | 24.4±0.0 | 24.4±0.0 |  | 23.7±0.0 | 23.7±0.0 | 23.6±0.0 | 23.4±0.0 |
| BMI category | <18.5 | 1.3 | 1.4 | 1.1 | 1.2 |  | 1.7 | 2.0 | 2.0 | 2.7 |
|  | 18.5-22.9 | 29.0 | 27.9 | 28.9 | 28.9 |  | 38.7 | 42.5 | 44.8 | 47.1 |
|  | 23.0-24.9 | 31.3 | 30.2 | 29.6 | 29.9 |  | 28.1 | 26.4 | 26.7 | 25.1 |
| BMI | ≥25.0 | 38.4 | 40.5 | 40.3 | 40 |  | 31.4 | 29.1 | 26.5 | 25.1 |
| Elevated ALT | | 12.2 | 13.1 | 13.6 | 13.2 |  | 5.4 | 5.0 | 5.1 | 4.9 |
| Anemia |  | 2.6 | 2.5 | 2.2 | 2.3 |  | 8.9 | 10.2 | 10.3 | 10.4 |
| Energy intake, kcal/day | | 1672 ±5 | 1787±5 | 1904±4 | 2004±4 |  | 1525±3 | 1632±3 | 1745±3 | 1814±3.4 |
| UPF intake, median (IQR) | | 1.9 (1.2-2.5) | 4.5 (3.7-5.2) | 7.8 (6.8-8.8) | 13.7 (11.6-17.3) | | 1.9 (1.1-2.5) | 4.6 (3.8-5.3) | 8.1 (7.1-9.17) | 14 (11.9-17.4) |
| NOVA 1, median (IQR) | | 96.4 (94.6-98.0) | 93.0 (91.1-94.7) | 89.3 (87.1-91.2) | 82.2 (77.6-85.4) | | 97.1 (95.3-98.9) | 93.7 (91.8-95.6) | 89.8 (87.6-92.0) | 82.7 (78.2-86.1) |
| **Prevalent disease at baseline** | |  |  |  |  |  |  |  |  |  |
| Cancer |  | 2.2 | 2.0 | 2.1 | 2.2 |  | 4.0 | 3.7 | 3.5 | 3.7 |
| MI |  | 3.4 | 2.6 | 2.5 | 2.2 |  | 1.6 | 1.2 | 1.2 | 1.0 |
| CPD |  | 0.6 | 0.6 | 0.6 | 0.6 |  | 0.7 | 0.6 | 0.6 | 0.6 |
| CKD |  | 6.1 | 5.9 | 6.1 | 6.3 |  | 3.5 | 2.8 | 2.8 | 3.0 |
| High blood glucose | | 14.2 | 11.2 | 10.0 | 8.5 |  | 9.0 | 5.8 | 4.8 | 3.9 |
| High blood pressure | | 56.6 | 53.3 | 50.1 | 48.5 |  | 43.6 | 37.6 | 35.2 | 32.6 |
| Metabolic syndrome | | 22.5 | 21.2 | 19.2 | 17.9 |  | 24.7 | 19.2 | 16.9 | 14.4 |
| Stroke |  | 1.1 | 0.8 | 0.8 | 1.0 |  | 0.5 | 0.4 | 0.4 | 0.3 |
| Disease score | 1 | 4.5 | 3.9 | 3.8 | 3.3 |  | 2.9 | 2.3 | 2.1 | 2.0 |
|  | ≥2 | 9.0 | 7.8 | 7.9 | 7.9 |  | 7.5 | 6.3 | 6.2 | 6.7 |
| Postmenopausal women | | |  |  |  |  | 73.6 | 61.0 | 58.5 | 57.1 |
| Oral contraceptives user | | | |  |  |  | 82.5 | 83.2 | 84.1 | 83.4 |

Values are % or means ±SE unless indicated otherwise. Details are shown in S2 Table

Mean BMI was adjusted for age and the means of dietary variables were adjusted for age and total energy intake.

AMI, Total activity Metabolic Index; ALT, Alanine amino-transferase, BMI, body mass index; MI, myocardial infarction; CPD, chronic pulmonary diseases; CKD, chronic kidney disease, NOVA1, Unprocessed/minimally processed foods
