# Supplementary material for: The intake of ultra-processed foods, all-cause, cancer and cardiovascular mortality in the Korean Genome and Epidemiology Study-Health Examinees (KoGES-HEXA) cohort
Source: PLoS One. 2023 May 4;18(5):e0285314. doi: 10.1371/journal.pone.0285314 (PMC10159145; doi:10.1371/journal.pone.0285314)
Supplement: S3 Table — (DOCX) [file pone.0285314.s003.docx]

# S3 Table. Hazard ratios and 95% CI of all-cause, cancer and CVD mortality according to quartiles of UPF intake

|  | **Quartiles of UPF intake (% of food weight)** | | | | | | | | | |
| --- | --- | --- | --- | --- | --- | --- | --- | --- | --- | --- |
|  |  | **Men** |  |  |  |  | **Women** |  |  |  |
|  | **Q1** | **Q2** | **Q3** | **Q4** | ***P*-trend*** | **Q1** | **Q2** | **Q3** | **Q4** | **P-trend*** |
| **All-cause mortality** |  |  |  |  |  |  |  |  |  |  |
| Events | 637 | 497 | 465 | 467 |  | 459 | 337 | 297 | 297 |  |
| Person years | 102,890 | 102,362 | 102,273 | 102,340 |  | 200,521 | 198,509 | 198,438 | 197,989 |  |
| Model 1 | 1.00 | 0.98 (0.87-1.1) | 1.00 (0.88-1.13) | 1.06 (0.94-1.2) | 0.547 | 1.00 | 0.97 (0.84-1.12) | 0.91 (0.78-1.05) | 0.94 (0.81-1.09) | 0.547 |
| Model 2 | 1.00 | 0.98 (0.87-1.1) | 1.04 (0.92-1.17) | 1.10 (0.97-1.25) | 0.516 | 1.00 | 0.98 (0.85-1.13) | 0.93 (0.80-1.08) | 0.96 (0.82-1.12) | 0.862 |
| Model 3 | 1.00 | 0.98 (0.87-1.11) | 1.04 (0.92-1.17) | 1.10 (0.97-1.24) | 0.564 | 1.00 | 0.98 (0.85-1.13) | 0.93 (0.80-1.08) | 0.96 (0.82-1.12) | 0.850 |
| Model 4 | 1.00 | 0.98 (0.87-1.10) | 1.03 (0.91-1.16) | 1.08 (0.95-1.22) | 0.564 | 1.00 | 0.98 (0.85-1.13) | 0.92 (0.80-1.07) | 0.95 (0.81-1.11) | 0.950 |
| **Cancer mortality** |  |  |  |  |  |  |  |  |  |  |
| Events | 298 | 236 | 214 | 204 |  | 233 | 199 | 161 | 175 |  |
| Model 1 | 1.00 | 1.00 (0.84-1.19) | 0.99 (0.83-1.19) | 1.01 (0.84-1.21) | 0.958 | 1.00 | 1.08 (0.90-1.31) | 0.92 (0.75-1.13) | 1.04 (0.85-1.27) | 0.958 |
| Model 2 | 1.00 | 0.99 (0.84-1.18) | 1.02 (0.85-1.22) | 1.03 (0.85-1.24) | 0.862 | 1.00 | 1.08 (0.89-1.31) | 0.92 (0.75-1.13) | 1.03 (0.84-1.26) | 0.913 |
| Model 3 | 1.00 | 0.99 (0.84-1.18) | 1.02 (0.85-1.22) | 1.03 (0.85-1.24) | 0.879 | 1.00 | 1.08 (0.89-1.31) | 0.92 (0.75-1.13) | 1.03 (0.84-1.27) | 0.926 |
| Model 4 | 1.00 | 0.99 (0.83-1.18) | 1.01 (0.84-1.2) | 1.02 (0.84-1.22) | 0.950 | 1.00 | 1.08 (0.89-1.31) | 0.92 (0.74-1.13) | 1.02 (0.83-1.26) | 0.950 |
| **CVD mortality** |  |  |  |  |  |  |  |  |  |  |
| Events | 108 | 83 | 76 | 66 |  | 85 | 37 | 47 | 37 |  |
| Model 1 | 1.00 | 0.95 (0.71-1.27) | 0.94 (0.7-1.27) | 0.86 (0.63-1.18) | 0.547 | 1.00 | 0.63 (0.43-0.93) | 0.87 (0.61-1.26) | 0.73 (0.49-1.09) | 0.547 |
| Model 2 | 1.00 | 0.97 (0.73-1.30) | 1.00 (0.74-1.35) | 0.91 (0.66-1.25) | 0.862 | 1.00 | 0.67 (0.45-0.99) | 0.95 (0.66-1.38) | 0.81 (0.54-1.21) | 0.862 |
| Model 3 | 1.00 | 0.97 (0.73-1.29) | 0.99 (0.73-1.34) | 0.90 (0.66-1.25) | 0.850 | 1.00 | 0.67 (0.45-0.99) | 0.96 (0.66-1.38) | 0.81 (0.54-1.21) | 0.850 |
| Model 4 | 1.00 | 0.96 (0.72-1.28) | 0.98 (0.72-1.32) | 0.88 (0.64-1.22) | 0.950 | 1.00 | 0.67 (0.45-0.99) | 0.95 (0.65-1.37) | 0.80 (0.53-1.19) | 0.950 |

Values are HR (95% CI) unless indicated otherwise; * False discovery rate adjusted.

Model 1. Adjusted for age and total energy intake.

Model 2. Adjusted for age, total energy intake, education level, monthly income, marital status, smoking, drinking, and regular physical exercise.

Model 3 was further adjusted for BMI

Model 4 was representative of model 3 + comorbidity score, menopausal status, use of oral contraceptives and hypercholesterolemia for CVD mortality.
