# Supplementary material for: The intake of ultra-processed foods, all-cause, cancer and cardiovascular mortality in the Korean Genome and Epidemiology Study-Health Examinees (KoGES-HEXA) cohort
Source: PLoS One. 2023 May 4;18(5):e0285314. doi: 10.1371/journal.pone.0285314 (PMC10159145; doi:10.1371/journal.pone.0285314)
Supplement: S4 Table — (DOCX) [file pone.0285314.s004.docx]

# S4 Table. Association of UPF items/ subgroups and all-cause mortality

|  | **Quartiles of UPF items/sub-groups** | | | | | | | | |
| --- | --- | --- | --- | --- | --- | --- | --- | --- | --- |
|  |  | **Men** |  |  |  |  | **Women** |  |  |
|  | **Q1** | **Q2** | **Q3** | **Q4** |  | **Q1** | **Q2** | **Q3** | **Q4** |
|  | HR (95% CI)^1^ | HR (95% CI) | HR (95% CI) | HR (95% CI) |  | HR (95% CI) | HR (95% CI) | HR (95% CI) | HR (95% CI) |
| Instant noodles | 1.00 | 1.06 (0.94-1.19) | 0.94 (0.83-1.07) | 1.04 (0.92-1.18) |  | 1.00 | 0.85 (0.59-1.22) | 0.94 (0.81-1.08) | 1.05 (0.92-1.21) |
| Breads | 1.00 | 0.88 (0.77-1.00) | 0.99 (0.89-1.11) | 0.96 (0.85-1.08) |  | 1.00 | 0.93 (0.79-1.08) | 1.00 (0.87-1.15) | 1.05 (0.9-1.23) |
| Bread spreads | 1.00 | 1.02 (0.92-1.13) |  |  |  | 1.00 | 0.941 (0.83-1.10) | 0.96 (0.7-1.33) | 0.94 (0.82-1.07) |
| Breakfast cereals & snacks | 1.00 | 0.88 (0.77-1.03) | 0.96 (0.87-1.07) |  |  | 1.00 | 1.11 (0.46-2.67) | 1.10 (0.45-2.65) | 1.13 (0.47-2.75) |
| Candies and chocolate | 1.00 | 0.89 (0.76-1.04) | 0.95 (0.86-1.04) |  |  | 1.00 | 0.80 (0.69-0.93) | 0.92 (0.81-1.04) |  |
| Pizza and hamburger | 1.00 | 0.93 (0.82-1.06) |  |  |  | 1.00 | 0.93 (0.80-1.10) |  |  |
| Red meat and Fish | 1.00 | 1.15 (1.01-1.32) | 1.10 (0.98-1.23) | 1.26 (1.11-1.43) |  | 1.00 | 1.02 (0.86-1.21) | 1.21 (1.05-1.40) | 1.22 (1.05-1.43) |
| Milk | 1.00 | 0.97 (0.85-1.10) | 1.03 (0.90-1.17) | 1.13 (1.01-1.26) |  | 1.00 | 1.01 (0.86-1.19) | 0.90 (0.78-1.04) | 1.08 (0.93-1.25) |
| Yoghurt | 1.00 | 1.04 (0.9-1.20) | 0.91 (0.82-1.02) | 1.05 (0.93-1.18) |  | 1.00 | 0.93 (0.81-1.08) | 0.87 (0.74-1.01) | 0.88 (0.77-1.02) |
| Ice cream | 1.00 |  | 0.90 (0.80-1.02) | 0.94 (0.83-1.06) |  | 1.00 |  | 0.87 (0.74-1.01) | 0.81 (0.69-0.94) |
| Coffee creamer | 1.00 | 1.02 (0.89-1.17) | 0.92 (0.83-1.03) | 1.00 (0.87-1.14) |  | 1.00 | 0.98 (0.70-1.37) | 0.83 (0.72-0.96) | 0.92 (0.81-1.04) |
| Soymilk drink | 1.00 | 1.04 (0.92-1.19) | 1.12 (1.00-1.25) |  |  | 1.00 | 1.02 (0.69-1.53) | 1.08 (0.96-1.21) |  |
| Soft drinks & fruit sodas | 1.00 | 0.92 (0.99-1.10) |  |  |  | 1.00 | 1.01 (0.88-1.16) |  |  |
| Sweet rice punch (“Sikhye”) | 1.00 | 0.81 (0.55-1.18) | 0.95 (0.86-1.05) | 0.95 (0.84-1.07) |  | 1.00 | 0.97 (0.64-1.47) | 1.04 (0.92-1.18) | 0.93 (0.81-1.07) |

1 Adjusted for age and total energy intake, education level, monthly income, marital status, smoking, alcohol consumption, and physical activity, BMI, comorbidity score and the prudent dietary pattern.
