# Supplementary material for: The intake of ultra-processed foods, all-cause, cancer and cardiovascular mortality in the Korean Genome and Epidemiology Study-Health Examinees (KoGES-HEXA) cohort
Source: PLoS One. 2023 May 4;18(5):e0285314. doi: 10.1371/journal.pone.0285314 (PMC10159145; doi:10.1371/journal.pone.0285314)
Supplement: S5 Table — (DOCX) [file pone.0285314.s005.docx]

# S5 Table. Association of ultra-processed food items/ subgroups and cancer-specific mortality

|  | Quartiles of UPF intake, % food weight | | | | | | | |
| --- | --- | --- | --- | --- | --- | --- | --- | --- |
|  |  | **Men** |  |  |  | Women |  |  |
|  | Q1 | Q2 | Q3 | Q4 |  | Q2 | Q3 | Q4 |
| **UPF subgroups** | HR (95% CI) | HR (95% CI) | HR (95% CI) | HR (95% CI) |  | HR (95% CI) | HR (95% CI) | HR (95% CI) |
| Instant noodles | 1.00 | 1.17 (0.99-1.38) | 1.08 (0.9-1.29) | 0.94 (0.78-1.14) |  | 0.68 (0.4-1.15) | 0.88 (0.72-1.07) | 1.01 (0.84-1.21) |
| Breads | 1.00 | 0.91 (0.75-1.1) | 1.12 (0.95-1.32) | 0.96 (0.8-1.16) |  | 1.16 (0.94-1.42) | 1.14 (0.94-1.38) | 1.21 (0.98-1.5) |
| Bread spreads | 1.00 | 1.01 (0.871.17) |  |  |  | 0.97 (0.82-1.15) |  |  |
| Breakfast cereals & snacks | 1.00 | 0.95 (0.77-1.17) | 1.03 (0.88-1.19) |  |  | 1.10 (0.89-1.35) | 1.11 (0.93-1.32) |  |
| Candies and chocolate | 1.00 | 1.04 (0.88-1.23) | 1.02 (0.88-1.2) |  |  | 0.88 (0.72-1.07) | 0.89 (0.75-1.06) |  |
| Pizza and hamburger | 1.00 | 0.87 (0.71-1.05) |  |  |  | 0.93 (0.77-1.13) |  |  |
| Meats and Fish | 1.00 | 1.11 (0.92-1.36) | 1.18 (1.01-1.40) | 1.18 (0.97-1.43) |  | 1.02 (0.82-1.27) | 1.26 (1.05-1.53) | 1.13 (0.92-1.39) |
| Milk | 1.00 | 1.11 (0.92-1.33) | 1.14 (0.94-1.38) | 1.13 (0.96-1.33) |  | 1.02 (0.82-1.27) | 0.83 (0.68-1) | 0.99 (0.82-1.21) |
| Yoghurt | 1.00 | 1.16 (0.94-1.43) | 1.00 (0.85-1.18) | 1.09 (0.92-1.3) |  | 1.05 (0.86-1.27) | 0.88 (0.71-1.08) | 0.87 (0.72-1.05) |
| Ice cream | 1.00 | 0.92 (0.81-1.10) |  |  |  | 0.85 (0.72-1.00) |  |  |
| Coffee creamer | 1.00 | 1.17 (0.96-1.43) | 1.04 (0.89-1.22) | 1.15 (0.94-1.4) |  | 1.17 (0.96-1.43) | 1.04 (0.89-1.22) | 1.15 (0.94-1.4) |
| Soymilk drink | 1.00 | 1.08 (0.89-1.3) | 1.07 (0.9-1.26) |  |  | 0.74 (0.4-1.39) | 1.11 (0.95-1.3) |  |
| Soft drinks & fruit sodas | 1.00 | 0.99 (0.86-1.15) |  |  |  | 0.91 (0.76-1.1) |  |  |

1 Adjusted for age and total energy intake, education level, monthly income, marital status, smoking, alcohol consumption, and physical activity, BMI, comorbidity score and the prudent dietary pattern.
