# Supplementary material for: The intake of ultra-processed foods, all-cause, cancer and cardiovascular mortality in the Korean Genome and Epidemiology Study-Health Examinees (KoGES-HEXA) cohort
Source: PLoS One. 2023 May 4;18(5):e0285314. doi: 10.1371/journal.pone.0285314 (PMC10159145; doi:10.1371/journal.pone.0285314)
Supplement: S6 Table — (DOCX) [file pone.0285314.s006.docx]

# S6 Table. Association of ultra-processed food items/ subgroups and CVD-specific mortality

|  | **Quartiles of UPF items/sub-groups** | | | | | | | |
| --- | --- | --- | --- | --- | --- | --- | --- | --- |
|  |  | **Men** |  |  |  | **Women** |  |  |
|  | Q1 | Q2 | Q3 | Q4 |  | Q2 | Q3 | Q4 |
| **UPF subgroups** | HR (95% CI)^1^ | HR (95% CI) | HR (95% CI) | HR (95% CI) |  | HR (95% CI) | HR (95% CI) | HR (95% CI) |
| Instant noodles | 1.00 | 0.94 (0.69-1.27) | 0.89 (0.64-1.22) | 1.23 (0.91-1.65) |  | 0.99 (0.41-2.44) | 0.9 (0.62-1.32) | 1.03 (0.72-1.49) |
| Breads | 1.00 | 0.85 (0.61-1.17) | 1.01 (0.77-1.34) | 0.85 (0.62-1.16) |  | 0.92 (0.63-1.34) | 0.83 (0.57-1.19) | 0.64 (0.4-1.03) |
| Bread spreads | 1.00 | 0.911 (0.70-1.17) |  |  |  | 0.78 (0.54-1.12) |  |  |
| Breakfast cereals & snacks | 1.00 | 0.86 (0.61-1.23) | 0.84 (0.65-1.09) |  |  | 0.74 (0.46-1.19) | 1.13 (0.81-1.58) |  |
| Candies and chocolate | 1.00 | 0.74 (0.54-1.02) | 0.83 (0.63-1.09) |  |  | 0.61 (0.39-0.97) | 1.11 (0.81-1.52) |  |
| Pizza and hamburger | 1.00 | 1.06 (0.78-1.45) |  |  |  | 0.87 (0.58-1.32) |  |  |
| Meats and Fish | 1.00 | 1.25 (0.91-1.73) | 0.99 (0.74-1.33) | 1.26 (0.92-1.72) |  | 1.08 (0.711-1.64) | 1.14 (0.78-1.65) | 1.19 (0.78-1.81) |
| Milk | 1.00 | 0.87 (0.64-1.2) | 0.91 (0.66-1.26) | 1.01 (0.77-1.33) |  | 1.31 (0.88-1.96) | 0.92 (0.63-1.33) | 1.18 (0.81-1.72) |
| Yoghurt | 1.00 | 0.92 (0.64-1.33) | 0.78 (0.59-1.03) | 0.96 (0.72-1.28) |  | 0.99 (0.69-1.43) | 0.86 (0.57-1.29) | 0.78 (0.54-1.14) |
| Ice cream | 1.00 | 0.90 (0.71-1.14) |  |  |  | 0.87 (0.63-1.21) |  |  |
| Coffee creamer | 1.00 | 0.94 (0.66-1.34) | 1.01 (0.78-1.3) | 0.96 (0.69-1.35) |  | 1.49 (0.73-3.07) | 0.73 (0.5-1.07) | 0.95 (0.68-1.32) |
| Soymilk drink | 1.00 | 1.07(0.85-1.35) |  |  |  | 1.10 (0.81-1.50) |  |  |
| Soft drinks & fruit sodas | 1.00 | 0.88 (0.68-1.12) |  |  |  | 1.36 (0.98-1.91) |  |  |
| Sweet rice punch (“Sikhye”) | 1.00 | 0.87 (0.36-2.12) | 0.74 (0.56-0.96) | 0.93 (0.69-1.24) |  | 1.69 (0.69-4.16) | 1.17 (0.85-1.61) | 0.96 (0.66-1.41) |

1 Adjusted for age and total energy intake, education level, monthly income, marital status, smoking, alcohol consumption, and physical activity, BMI, comorbidity score and the prudent dietary pattern.
