# Supplementary material for: The intake of ultra-processed foods, all-cause, cancer and cardiovascular mortality in the Korean Genome and Epidemiology Study-Health Examinees (KoGES-HEXA) cohort
Source: PLoS One. 2023 May 4;18(5):e0285314. doi: 10.1371/journal.pone.0285314 (PMC10159145; doi:10.1371/journal.pone.0285314)
Supplement: S7 Table — (DOCX) [file pone.0285314.s007.docx]

# S7 Table. Sensitivity analyses of the association between total UPF intake and all-cause mortality

|  | **Quartiles of UPF, % food weight** | | | | | | | | | | | |
| --- | --- | --- | --- | --- | --- | --- | --- | --- | --- | --- | --- | --- |
|  | **Men** | | | | |  | **Women** | | | | | |
|  | **Q1** | **Q2** | **Q3** | **Q4** | **P _for trend_** |  |  | | **Q2** | **Q3** | **Q4** | **P _for trend_** |
| **Adjusted for Unprocessed/minimally processed foods food intake** | | | | | | | | | |  |  |  |
| HR (95% CI) | 1.00 | 0.94 (0.82-1.08) | 0.92 (0.78-1.08) | 0.85 (0.7-1.04) | 0.124 |  |  | 0.99 (0.84-1.16) | | 0.93 (0.77-1.13) | 1.00 (0.79-1.27) | 0.97 |
| **Adjusted for LTPA** | | | | | | | | | | | | |
| HR (95% CI) | 1.00 | 0.98 (0.87-1.11) | 1.03 (0.91-1.17) | 1.09 (0.96-1.23) | 0.129 |  |  | 0.98 (0.85-1.14) | | 0.93 (0.8-1.08) | 0.95 (0.82-1.11) | 0.56 |
| **Adjusted for alcohol intake (g/day)** | | | | | | | | | | | | |
| HR (95% CI) | 1.00 | 0.99 (0.88-1.12) | 1.04 (0.92-1.18) | 1.10 (0.97-1.25) | 0.095 |  |  | 0.98 (0.85-1.13) | | 0.92 (0.79-1.07) | 0.95 (0.81-1.1) | 0.42 |
| **Excluding participants who died before 5 years of follow-up** | | | | | | | | | | | | |
| HR (95% CI) | 1.00 | 1.01 (0.88-1.16) | 1.05 (0.87-1.17) | 1.07 (0.93-1.25) | 0.342 |  |  | 1.02 (0.87-1.22) | | 0.96 (0.80-1.14) | 0.95 (0.79-1.14) | 0.475 |
| **Excluding participants with missing data on confounders** | | | | | | | | | |  |  |  |
| HR (95% CI) | 1.00 | 0.95 (0.84-1.08) | 1.01 (0.89-1.15) | 1.08 (0.94-1.23) | 0.112 |  |  | 1.03 (0.89-1.2) | | 0.92 (0.79-1.09) | 0.95 (0.8-1.12) | 0.43 |
| **Excluding participants who died from accidents** | | | | | | | | | | |  |  |
| HR (95% CI) | 1.00 | 0.98 (0.87-1.11) | 1.01 (0.89-1.15) | 1.03 (0.9-1.17) | 0.635 |  |  | 0.98 (0.84-1.14) | | 0.93 (0.79-1.09) | 0.99 (0.84-1.16) | 0.83 |

Models were adjusted for age and total energy intake, education level, monthly income, marital status, smoking, alcohol consumption, and physical activity, BMI, comorbidity score, menopausal status, and use of oral contraceptives.
