# Supplementary figures and images for: The intake of ultra-processed foods, all-cause, cancer and cardiovascular mortality in the Korean Genome and Epidemiology Study-Health Examinees (KoGES-HEXA) cohort
Source: PLoS One. 2023 May 4;18(5):e0285314. doi: 10.1371/journal.pone.0285314 (PMC10159145; doi:10.1371/journal.pone.0285314)

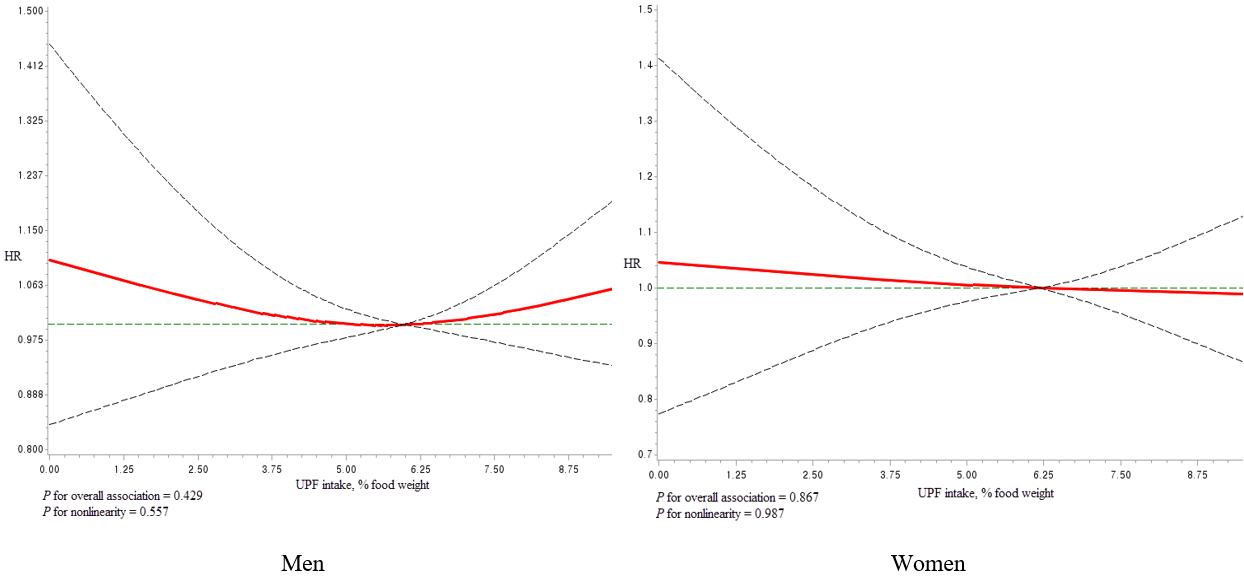

Supplement: S1 Fig — The red line indicates hazard ratios (HR), and the dotted lines indicate the 95% CI. The reference value for HRs was 5.6 (median of % food weight from UPF). The model was adjusted for age, total energy intake, education level, monthly income, marital status, smoking, drinking, regular physical exercise, comorbidity score, menopausal status, and use of oral contraceptives. (TIF) [file pone.0285314.s008.tif]

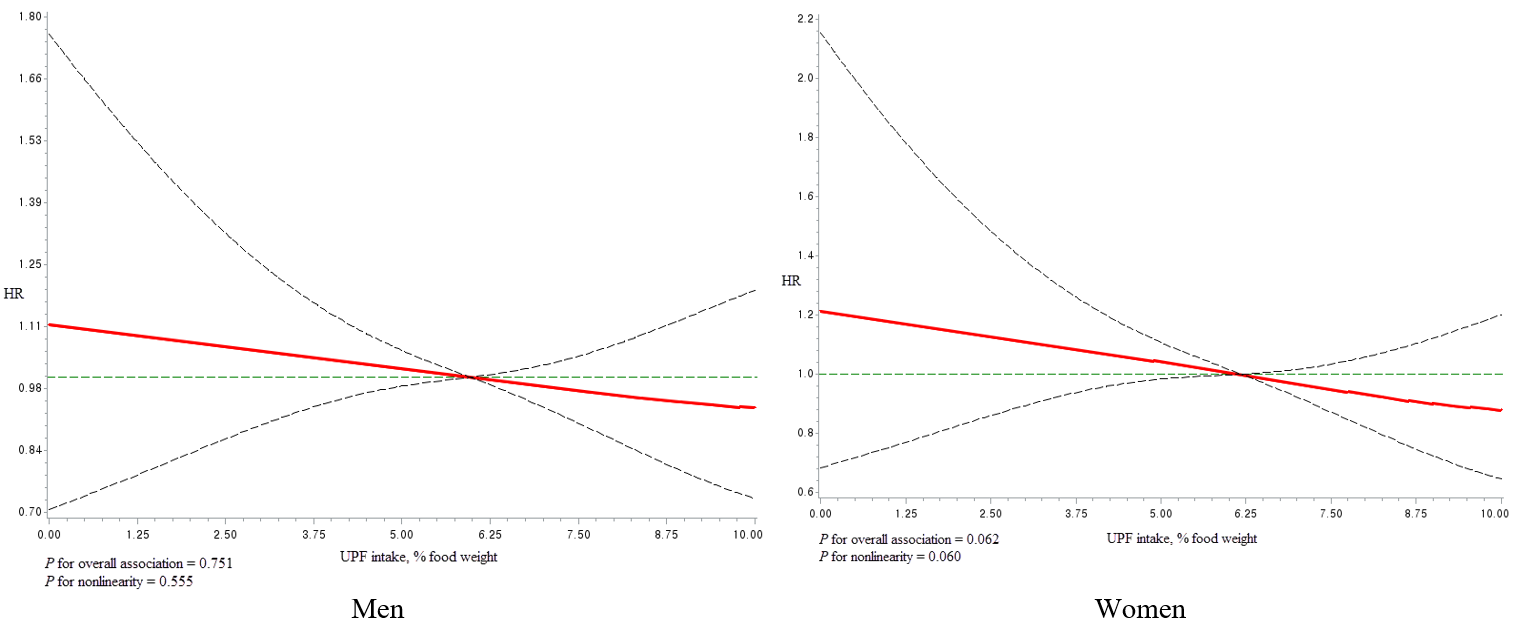

Supplement: S2 Fig — The red line indicates hazard ratios (HR), and the dotted lines indicate the 95% CI. The reference value for HRs was 5.6 (median of % food weight from UPF). The model was adjusted for age, total energy intake, education level, monthly income, marital status, smoking, drinking, regular physical exercise, comorbidity score, menopausal status, use of oral contraceptives, and hypercholesterolemia. (TIF) [file pone.0285314.s009.tif]

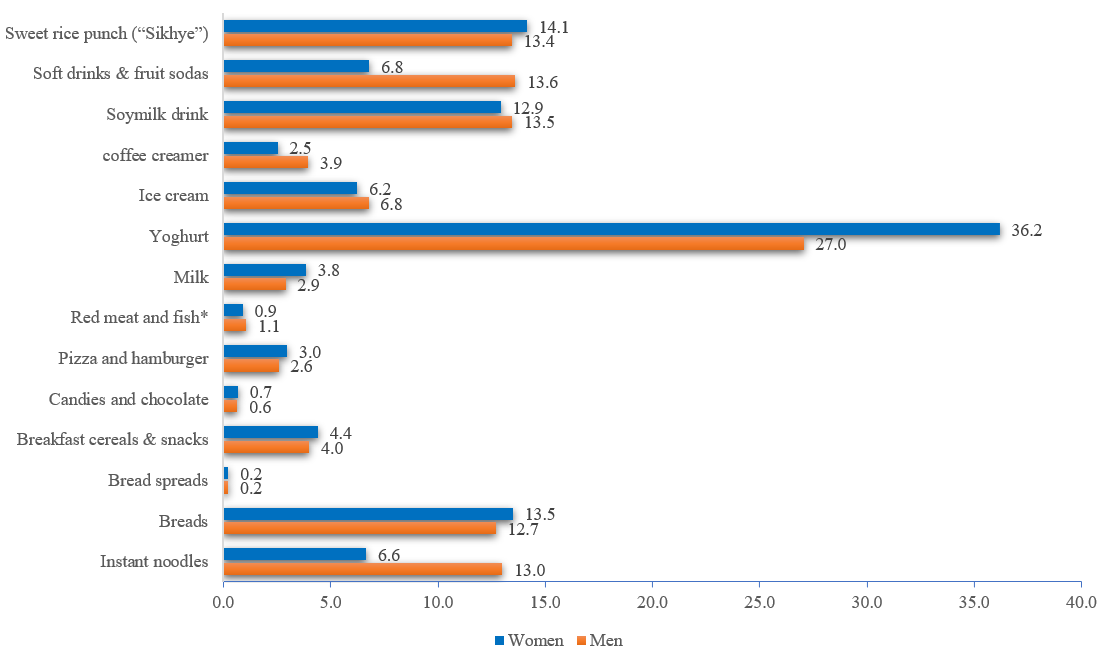

Supplement: S3 Fig — (TIF) [file pone.0285314.s010.tif]
